# Low-Grade Appendiceal Mucinous Neoplasm in a Patient With Ulcerative Colitis Detected on Routine Surveillance Colonoscopy: A Case Report and Review of Literature

**DOI:** 10.7759/cureus.85844

**Published:** 2025-06-12

**Authors:** Yantong Huang, Ronen Arai, Brett Staller

**Affiliations:** 1 Osteopathic Medicine, Nova Southeastern University Dr. Kiran C. Patel College of Osteopathic Medicine, Davie, USA; 2 Gastroenterology, Broward Health, Coral Springs, USA; 3 Diagnostic Radiology, Broward Health, Fort Lauderdale, USA

**Keywords:** appendicular mucocele, mucocele of the appendix, neoplasm of the appendix, rare lesions in the appendix, ulcerative colitis

## Abstract

Appendiceal mucocele (AM) is characterized by the dilation of the appendix due to the accumulation of mucinous material within its lumen, often discovered incidentally on imaging. Symptoms of AM are typically nonspecific or absent, but patients may present with clinical features mimicking appendicitis or an adnexal mass. Ultrasound and CT imaging of the abdomen and pelvis are key in establishing the diagnosis. Early diagnosis is crucial, as a neoplastic mucocele can rupture, leading to pseudomyxoma peritonei (PMP), a clinical syndrome characterized by diffuse intra-abdominal gelatinous ascites with mucinous peritoneal involvement. While patients with ulcerative colitis (UC) are at increased risk for colorectal cancer, appendiceal neoplasms are rarely reported in this group. This case presentation involves a 70-year-old female patient with a history of UC who presented for a routine surveillance colonoscopy in the fall of 2024. She was diagnosed with mild UC in 2006 and had been in clinical remission since December 2018, and a colonoscopy performed in 2021 showed no mucosal inflammation. However, during the most recent surveillance colonoscopy in 2024, the appendiceal orifice was noted to be bulging, with mucus extruding, suspicious of a mucinous appendiceal neoplasm. Cold forceps biopsies were obtained, and histology showed only inflammatory changes in the appendiceal orifice. A contrast-enhanced CT of the abdomen and pelvis revealed a 3.2 x 2.0 cm tubular cystic structure originating from the base of the cecum, consistent with an AM, without signs of rupture or nodularity. The patient underwent a robotic-assisted partial cecectomy and appendectomy, and pathology confirmed a well-differentiated, low-grade appendiceal mucinous neoplasm (LAMN).

## Introduction

Appendiceal mucocele (AM) refers to the dilation of the appendix due to mucin accumulation. AMs are rare lesions that occur slightly more frequently in women and are commonly diagnosed in individuals in their fifth or sixth decade of life [1]. AM lesions are broadly divided into nonneoplastic or neoplastic epithelial types, and the neoplastic types are further categorized according to histological characteristics. Nonneoplastic mucinous lesions, such as simple mucoceles or retention cysts, typically result from obstructive processes that lead to epithelial degeneration without neoplastic transformation or hyperplasia. Neoplastic mucinous lesions consist of serrated polyps of the appendix, appendiceal mucinous neoplasms (AMNs), and mucinous adenocarcinoma of the appendix. Definitive diagnosis relies on a thorough histopathological examination of the entire appendix, particularly identifying epithelial invasion of the appendiceal wall, which is critical for distinguishing adenocarcinomas from other mucinous lesions [2].

AMs are often detected incidentally during imaging or endoscopic procedures for unrelated clinical concerns. Patients are usually asymptomatic or have nonspecific abdominal pain, but can present with right lower quadrant abdominal pain resembling acute appendicitis. In rarer cases, clinical presentation may include intermittent cramping abdominal pain, gastrointestinal bleeding related to mucocele-associated intussusception, genitourinary symptoms due to right ureter obstruction, or an acute abdomen due to rupture of the mucocele [3]. Additionally, the rupture of AMNs, whether spontaneously or intra-operatively, can lead to pseudomyxoma peritonei (PMP), characterized by the intraperitoneal spread of mucin and/or epithelial cells [4]. The mucus may accumulate in the pelvis, greater omentum, and perihepatic spaces, resulting in life-threatening complications such as compression of organs, intestinal obstruction, and cachexia [5].

Patients with a history of chronic inflammatory bowel disease (IBD), including ulcerative colitis (UC) and Crohn’s disease involving the colon, are routinely screened for colorectal cancer via colonoscopy. Patients with IBD should follow the surveillance protocol of an initial screening colonoscopy within eight to 10 years of disease diagnosis, followed by a surveillance colonoscopy every one to five years, depending on individual risk factors for colorectal cancer. The goal is to discover epithelial dysplasia early, before the development of colorectal cancer, and to reduce the morbidity and mortality associated with IBD-associated colorectal cancer [6]. The relationship between AM and IBD is not well-defined.

## Case presentation

**Figure 1:**
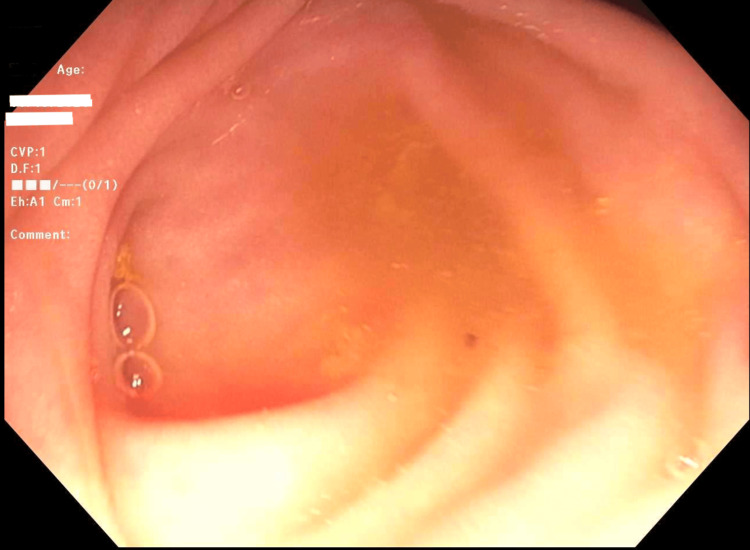
Appendiceal orifice from colonoscopy (2021).

A 70-year-old female patient with a history of UC presented for a routine surveillance colonoscopy in the fall of 2024. She was diagnosed with mild UC in 2006 and initially did not require medication for maintenance of remission. In 2012, she had a flare of proctosigmoiditis, requiring treatment with azathioprine. She stopped azathioprine in 2018 after presenting with bilateral pneumonitis with a positive *Cladosporidium* blood culture. Since then, she has been maintained on daily mesalamine and has been asymptomatic. A colonoscopy in December 2018 demonstrated mucosal remission. Her past medical history was significant for chronic gastroesophageal reflux disease (GERD) with a hiatal hernia, colonic diverticulosis, and hemorrhoids. Her family history was significant for colon cancer in her father. Past surgical history included a cholecystectomy and a dilation and curettage for a uterine polyp. Her last surveillance colonoscopy in 2021 was unremarkable, including a normal-appearing appendiceal orifice (Figure 1).

**Figure 2:**
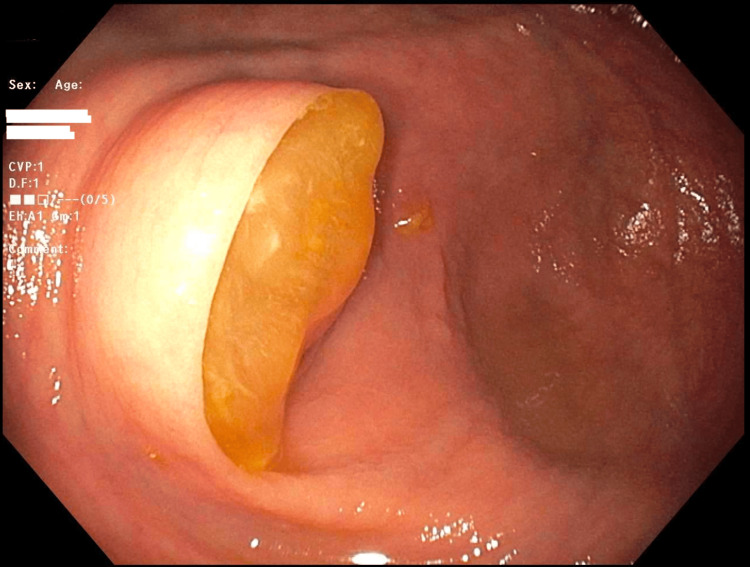
Bulging appendiceal orifice with extruding mucus from colonoscopy (2024).

**Figure 3:**
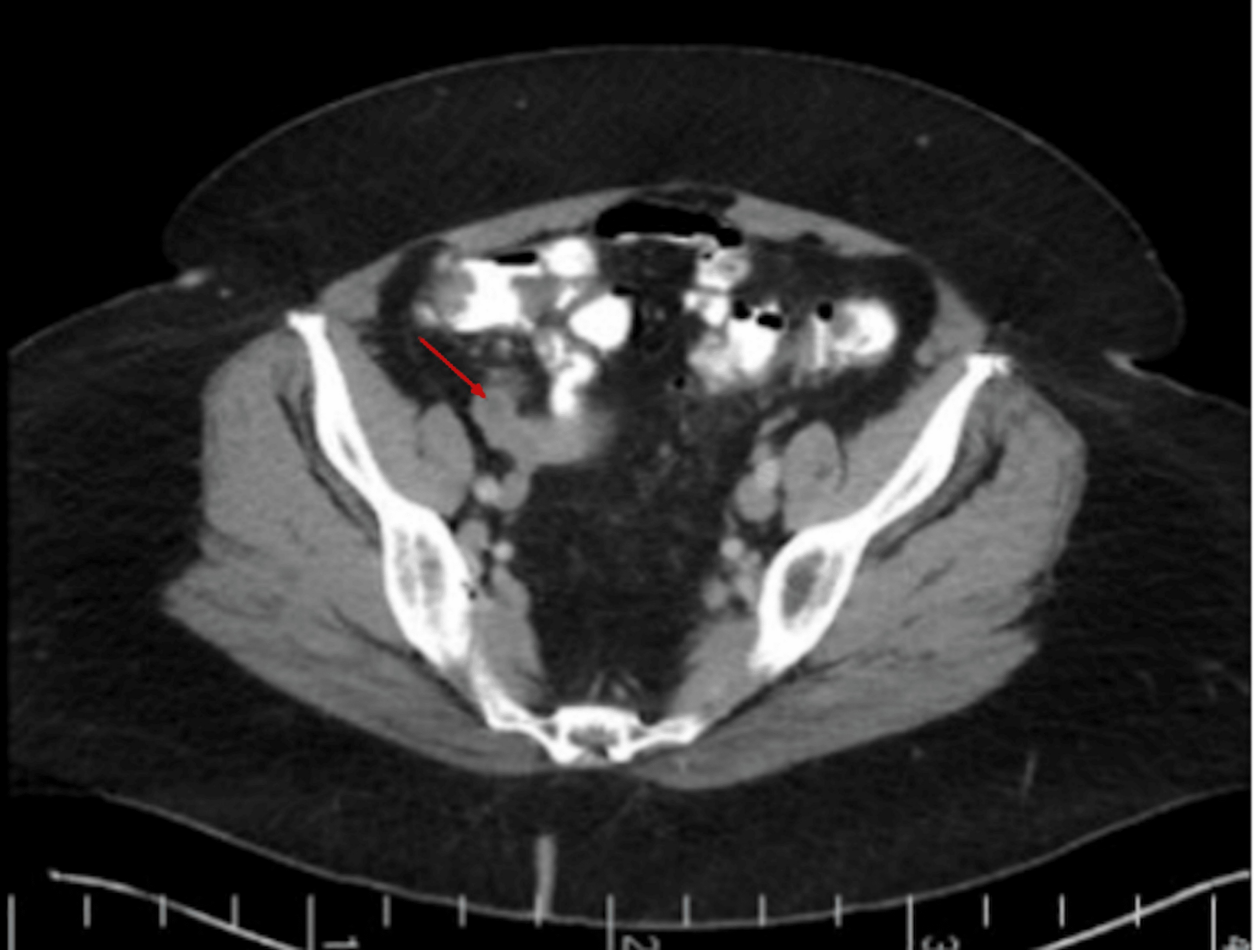
Axial CT image at the level of the pelvis; the red arrow demonstrates a round cystic lesion that originated from the cecum (post-colonoscopy, 2024). No associated nodularity and no evidence of rupture.

**Figure 4:**
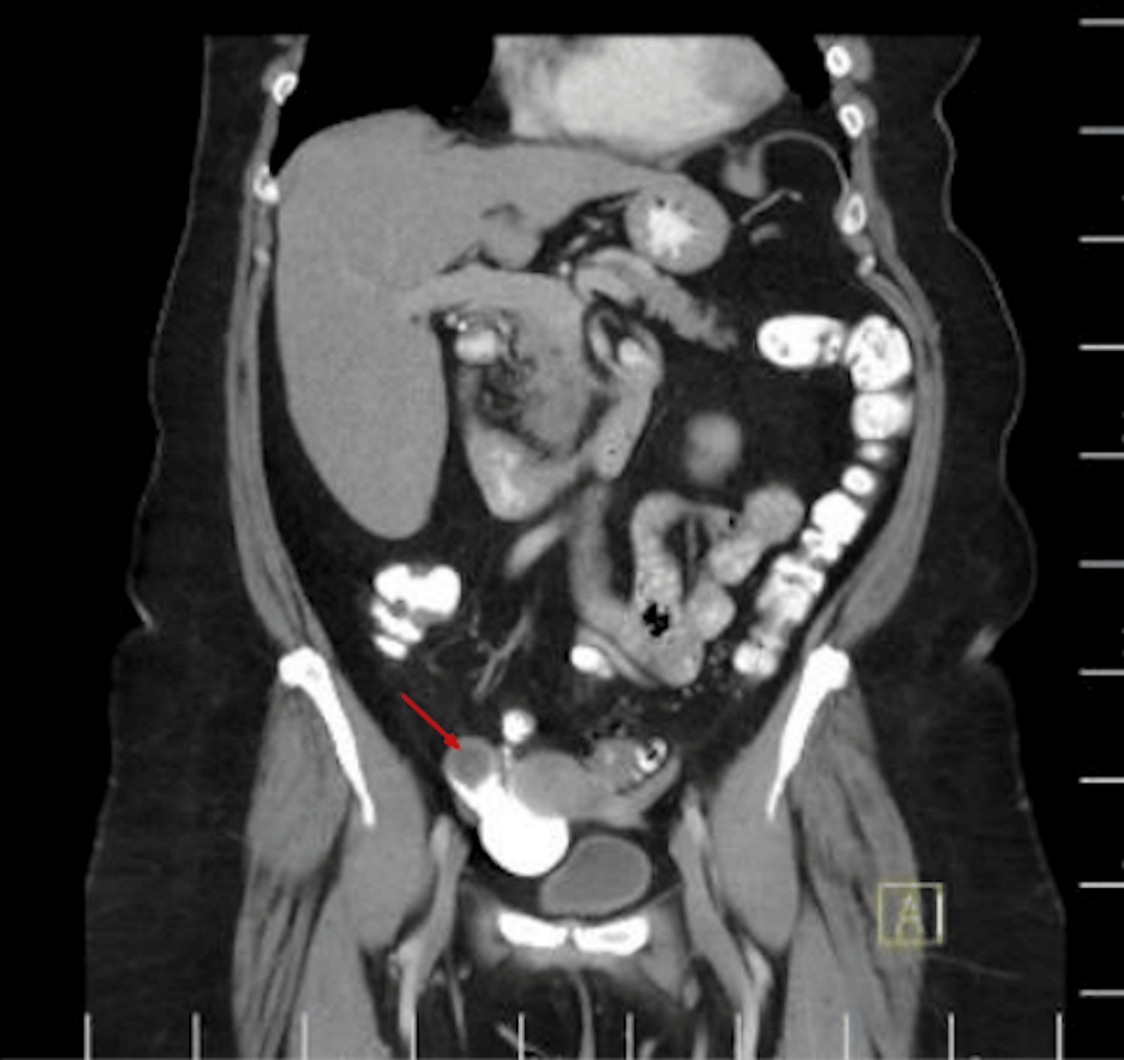
Coronal CT of the abdomen and pelvis; the red arrow demonstrates an oval cystic mass in the right lower quadrant with circumferential wall enhancement (post-colonoscopy, 2024).

Because of her history of UC, she continues to obtain routine colonoscopies every three years. At the time of presentation for her routine colonoscopy in 2024, she did not complain of recent flare-ups, including a lack of abdominal pain, weight loss, irregular bowel movements, bloody diarrhea, or cramping. However, during the surveillance colonoscopy in 2024, the appendiceal orifice was noted to be bulging, with mucus extruding, which was suspicious for a mucinous appendiceal neoplasm (Figure 2). Cold forceps biopsy was performed to make a definitive histological diagnosis prior to anticipated surgical resection; however, histopathology demonstrated only inflammatory changes in the appendiceal orifice. The rest of the colonoscopy was unremarkable, with no evidence of mucosal inflammation. A contrast-enhanced CT of the abdomen and pelvis revealed a 3.2 x 2.0 cm tubular cystic structure originating from the base of the cecum, consistent with an AM, without signs of rupture or nodularity (Figures 3, 4). The patient underwent robotic-assisted partial cecectomy and appendectomy. The appendix measured 4 x 0.4 cm, and pathology confirmed a well-differentiated, grade 1, low-grade AMN (LAMN) characterized by mildly enlarged hyperchromatic nuclei and low mitotic activity. No tumor invasion was seen, and proximal margins were negative for invasive carcinoma. Her post-operative course was unremarkable.

## Discussion

AM refers to a rare group of lesions that dilate the appendiceal lumen due to mucin accumulation. AMs are classified as nonneoplastic or neoplastic epithelial lesions, and the neoplastic lesions are further subcategorized according to histology. Nonneoplastic mucinous lesions, such as simple mucoceles or retention cysts, result from epithelial degeneration caused by luminal obstruction without evidence of mucosal hyperplasia or neoplastic change. Contributing factors include inflammatory conditions like appendicitis, fecalith or appendicolith-induced blockage, endometriosis involving the appendix, or other chronic local processes that result in scarring. In such cases, there is no predisposition toward neoplastic transformation.

Neoplastic mucinous lesions include serrated polyps, AMNs, and mucinous adenocarcinomas. Serrated polyps of the appendix, with or without dysplasia, morphologically resemble their colonic counterparts and typically lack features of fibrosis, appendiceal wall thickening, or loss of the lamina propria [2]. AMNs are dysplastic mucin-secreting tumors that extend into the muscularis mucosae but remain confined by the muscularis propria. These tumors exhibit a noninfiltrative growth pattern, with no destructive invasion or stromal desmoplasia. Based on the degree of dysplasia, AMNs are classified as either low-grade (LAMN) or high-grade (HAMN).

LAMNs are described as a true mucin-producing neoplasm with dysplastic epithelium and express a growth with a “pushing” border, sometimes leading to mural fibrosis or loss of muscular wall integrity [2]. Histologically, LAMNs are distinguished by the absence of epithelial invasion of the appendiceal wall, confined by the muscularis propria, and display mildly enlarged, hyperchromatic nuclei with low mitotic activity. HAMNs, while sharing similar features to LAMNs, show more pronounced dysplastic features. These include cribriform growth, full-thickness nuclear stratification, epithelial crowding with high-grade cytologic features, pleomorphic and hyperchromatic nuclei, and abundant atypical mitotic activity [2].

Although LAMNs and HAMNs are not invasive, their progressive expansion can exert pressure on and thin the appendiceal wall, which can rupture the appendix, resulting in spillage of cellular or acellular mucin into the peritoneal cavity, leading to PMP. This is a dreaded complication because the mucus-producing cells continue to proliferate while producing mucus, potentially progressing to the compression of organs, intestinal obstruction, and cachexia. The optimal treatment of PMP is unknown due to its rarity, which makes large-scale randomized clinical trials challenging and limits the validation of new therapies [7]. Treatment varies between institutions based on the severity of presentation and goals of therapy; some choose a more conservative approach via periodic surgical debulking for symptomatic relief, while others pursue a more aggressive approach of cytoreduction and heated intraperitoneal chemotherapy for cure. In asymptomatic patients with limited peritoneal disease or low-grade mucinous tumors that underwent resection, expectant observation is reasonable [8]. In symptomatic patients, treatments include periodic surgical debulking by resecting gross disease to limit mucus buildup and its pressure-like effect, to more aggressive approaches such as cytoreduction to remove all intra-abdominal and peritoneal disease, followed by heated intraperitoneal chemotherapy with agents such as mitomycin [9].

Mucinous adenocarcinomas are defined by the presence of invasive glandular structures exhibiting high-grade cytologic atypia, along with extracellular mucin comprising more than half of the lesion's cross-sectional area [10]. Histologically, these tumors are characterized by the formation of irregular or budding glands embedded in a desmoplastic stroma. This stroma is rich in a proteoglycan extracellular matrix and populated by fibroblasts or myofibroblasts with vesicular nuclei [2]. Mucinous adenocarcinomas can be classified as well, moderately, or poorly differentiated, with signet ring cells indicating poor differentiation [1].

AMs are frequently identified incidentally during imaging or endoscopic procedures for unrelated symptoms. Typical endoscopic findings include a smooth indentation on the cecal lumen and a rounded, protruding mass rising from the appendiceal orifice. Endoscopic forceps biopsy rarely yields a definitive diagnostic histology. An abdominal CT or ultrasound generally confirms the presence of AM and excludes other etiologies. CT findings in a patient with AM may demonstrate low-attenuated, well-encapsulated cystic structures, either round or tubular, in the right lower quadrant near the cecum. A distinct focal dilatation at the distal end of the appendix, while the proximal portion appears normal, is usually linked to an underlying neoplastic AM. Additionally, mural curvilinear calcifications, eggshell appearance, a diameter of >2cm, and absence of periappendiceal stranding on CT strongly suggest an underlying appendiceal neoplasm [11].

Treatment of AMs primarily involves surgical excision of the affected appendix to achieve both a definitive diagnosis and therapeutic benefit [12]. Surgical removal will also prevent the possibility of rupturing neoplastic lesions, which can lead to PMP. For most localized appendiceal mucinous lesions, a standard appendectomy is performed, and a decision for additional resection is usually made intraoperatively. A small section of the cecum is often removed alongside the appendix without encroaching on the ileocecal valve. If the base of the appendix is involved, where clear margins cannot be achieved with stapling, then a partial cecectomy, ileocecectomy, or a right hemicolectomy may be performed. For patients with unruptured LAMN or HAMN contained within the appendix and completely excised through an appendectomy, further surgical intervention, such as right hemicolectomy, is unnecessary [13]. If a nonperforated LAMN of the appendix is removed, with no evidence of tumor rupture or mucin spillage, and pathological examination confirms no mucin outside the appendix, the risk of recurrence is negligible, and routine surveillance may not be required [14]. Similarly, for nonperforated HAMNs, appendectomy alone is adequate, provided the entire specimen has been thoroughly assessed to rule out invasive adenocarcinoma [13]. For the patient in our case presentation with a well-differentiated LAMN with proximal margins negative for invasive adenocarcinoma, an uncomplicated robotic-assisted partial cecectomy and appendectomy was performed. Additionally, due to the lack of tumor rupture or mucin outside the appendix, more complex surgical interventions such as ileocecectomy or right hemicolectomy were unnecessary. For future management, she will continue taking mesalamine to maintain UC remission and will undergo another surveillance colonoscopy in three years.

UC and Crohn’s disease involving the colon are associated with an increased risk for colorectal cancer. Small intestinal Crohn’s disease also has an increased risk for small bowel adenocarcinoma. However, the association of IBD with AMs is unclear. Orta et al. suggest that IBD with concurrent colorectal dysplasia or cancer is a risk factor for developing appendiceal cystadenomas, suggesting the tumor is a neoplastic complication of IBD [15]. Others, such as Matsumoto et al., suggest that obstruction of the appendiceal orifice might play a role in the development of AM from blockage due to inflammation in the setting of IBD or an associated colorectal neoplasm [16]. Although the incidence of appendiceal neoplasms in patients with IBD is low, further research is needed to compare AMN in patients with and without IBD to determine the predisposition of IBD patients to AMN. This case highlights the importance of multidisciplinary collaboration between gastroenterologists, radiologists, and surgical oncologists in managing IBD patients with appendiceal neoplasms found incidentally on imaging or endoscopy. Furthermore, the case reminds us that in IBD patients with long-standing disease undergoing surveillance examination of the colon, careful attention must be paid to unexpected findings. Careful visual inspection of the appendiceal orifice is an important part of the surveillance protocol of colonoscopy in IBD patients.

## Conclusions

AM is a rare group of lesions characterized by a distended, mucus-filled, or ruptured appendix. Often diagnosed on imaging such as CT, endoscopy, or ultrasound, AMs are classified as either nonneoplastic or neoplastic epithelial lesions, and the neoplasias are further categorized according to histology. The relationship between IBD and AMs is unclear, and although the incidence of AMs in patients with IBD is low, the case reminds us of the importance of continued surveillance via colonoscopy and attention to unexpected findings. Furthermore, patients with IBD should continue to follow the dysplasia surveillance protocol of an initial screening colonoscopy within eight to 10 years of diagnosis, followed by a surveillance colonoscopy every one to five years.
